# Development and Modification of a Mobile Health Program to Promote Postpartum Weight Loss in Women at Elevated Risk for Cardiometabolic Disease: Single-Arm Pilot Study

**DOI:** 10.2196/16151

**Published:** 2020-04-09

**Authors:** Jacinda M Nicklas, Jenn A Leiferman, Steven Lockhart, Kristen M Daly, Sheana S Bull, Linda A Barbour

**Affiliations:** 1 Division of General Internal Medicine University of Colorado School of Medicine Aurora, CO United States; 2 Community and Behavioral Health Colorado School of Public Health Aurora, CO United States; 3 Adult and Child Consortium for Health Outcomes Research and Delivery Science University of Colorado School of Medicine Aurora, CO United States; 4 Division of Endocrinology, Metabolism and Diabetes University of Colorado School of Medicine Aurora, CO United States; 5 Division of Maternal Fetal Medicine University of Colorado School of Medicine Aurora, CO United States

**Keywords:** mobile health, postpartum, chronic disease, prevention, weight loss

## Abstract

**Background:**

Pregnancy complications in combination with postpartum weight retention lead to significant risks of cardiometabolic disease and obesity. The majority of traditional face-to-face interventions have not been effective in postpartum women. Mobile technology enables the active engagement of postpartum women to promote lifestyle changes to prevent chronic diseases.

**Objective:**

We sought to employ an interactive, user-centered, and participatory method of development, evaluation, and iteration to design and optimize the mobile health (mHealth) *Fit After Baby* program.

**Methods:**

For the initial development, a multidisciplinary team integrated evidence-based approaches for health behavior, diet and physical activity, and user-centered design and engagement. We implemented an iterative feedback and design process via 3 month-long beta pilots in which postpartum women with cardiometabolic risk factors participated in the program and provided weekly and ongoing feedback. We also conducted two group interviews using a structured interview guide to gather additional feedback. Qualitative data were recorded, transcribed, and analyzed using established qualitative methods. Modifications based on feedback were integrated into successive versions of the app.

**Results:**

We conducted three pilot testing rounds with a total of 26 women. Feedback from each pilot cohort informed changes to the functionality and content of the app, and then a subsequent pilot group participated in the program. We optimized the program in response to feedback through three iterations leading to a final version.

**Conclusions:**

This study demonstrates the feasibility of using an interactive, user-centered, participatory method of rapid, iterative design and evaluation to develop and optimize a mHealth intervention program for postpartum women.

**Trial Registration:**

ClinicalTrials.gov NCT02384226; https://www.clinicaltrials.gov/ct2/show/NCT02384226

## Introduction

### Pregnancy as a Stress Test to Expose Predilection to Chronic Disease

Certain pregnancy complications provide an early warning of future cardiometabolic risk [1]. Women with pregnancies complicated by gestational diabetes mellitus (GDM) have an approximately 50% increased risk for developing type 2 diabetes mellitus within 10 years, are likely to develop atherosclerosis earlier [2], and have an increased risk for hypertension [3] and cardiovascular disease (CVD) [4,5]. Preeclampsia, preterm delivery, delivery of a small-for-gestational age (SGA) neonate, hypertensive disorders in pregnancy, and GDM are independently associated with a 50%-300% increased risk for CVD [5]. About 30% of US women will have at least one of these predictive conditions during pregnancy [4].

### The Postpartum Period Is a Critical Window of Opportunity for Primary Prevention

Previous studies demonstrate that pregnancy weight retained beyond 6 to 12 months postpartum is usually retained long term and is a powerful independent risk factor for future obesity [6]. Given the significance of postpartum weight retention, the postpartum year is considered a critical window of opportunity to make lifestyle changes to decrease future risk of obesity and chronic disease [7-9]. Lifestyle changes, including weight loss, smoking cessation, improved diet, and physical activity can decrease the risk of diabetes and CVD [10-14]. Reducing postpartum weight retention also decreases the risk of weight-related complications in future pregnancies [15,16]. Unfortunately, most women do not return to their prepregnancy weight postpartum, and with each subsequent pregnancy, their risk of these obesity-related complications amplifies. However, previous studies demonstrate that postpartum women may be receptive to making lifestyle changes given their new awareness of risk factors that were unmasked during pregnancy as well as their motivation to create a healthy home for their offspring [17].

### A Lack of Available Treatment Options Tailored to Postpartum Mothers

Despite the importance and critical timing of the postpartum period, there are currently no clinically available evidence-based programs designed for overweight and obese postpartum women with recent pregnancy complications. Few lifestyle intervention studies have been conducted in postpartum women at elevated cardiometabolic risk. Studies attempting intensive face-to-face methodologies for weight loss and reduction in diabetes incidence similar to the successful Diabetes Prevention Program (DPP) demonstrate limited efficacy and poor retention in postpartum women [18-20]. This is due, at least in part, to multiple barriers to face-to-face participation described by postpartum women, including time constraints, infant and breastfeeding demands, older childcare responsibilities, and reluctance to spend time away from family [21,22]. Given these barriers, there is increasing interest in using technology to improve the efficacy of lifestyle interventions for this high-risk population [20,23].

### Use of Mobile Health

As of 2015, 82% of the US population aged 18 to 49 years owned an app-enabled mobile phone [24]. Women of childbearing age are one of the fastest growing user groups for mobile phones, across race and socioeconomic class. Mobile technology facilitates tracking of behavior and weight, allowing for real-time recording, feedback, and accountability. In light of escalating health care costs and rapid increases in the incidence of cardiometabolic disease, extending the reach of health promotion into daily life is an innovative approach for high-risk women with multiple and intensive family/work demands. However, despite the potential of this technology, the vast majority of available apps do not adequately include evidence-based strategies [25-27] or use behavioral theory [28,29], and almost none have been rigorously tested [30].

This study describes the iterative development process designed to optimize the mobile health (mHealth) *Fit After Baby* program. We employed an interactive user-centered and participatory method of rapid development, evaluation, and iteration [31-33]. We designed the *Fit After Baby* program to decrease obesity and risk factors for chronic disease by increasing postpartum weight loss, improving diet, and increasing physical activity.

## Methods

### Development of the Fit After Baby Program

We developed the mHealth *Fit After Baby* program using evidence-based strategies for weight loss, cardiometabolic disease prevention, and behavior change based on current evidence and best practices [34]. Guided by the Integrated Theory of mHealth [35], we incorporated a theoretical framework that included traditional health communication and behavioral theories including the *elaboration likelihood* model [36], the theory of planned behavior [37], the life-course approach [38,39], and Bandura’s model of social cognitive theory for behavioral change [40], as well as engagement strategies from design-thinking, user-centered design, and mobile technology in health promotion [41-45]. This inclusion of multiple and diverse theoretical perspectives in app design is critical to the creation of a mobile solution that is engaging, effective, and scalable. The content and structure were adapted from the DPP specifically for a postpartum population, and we incorporated features and techniques that have proven efficacy for weight loss such as self-monitoring, goal setting, remote coaching with tailored feedback, and social support [46-49]. We designed the *Fit After Baby* app to integrate with the commercial apps Fitbit and MyFitnessPal. Gamification through points and badges was used to motivate behaviors through accountability and reinforcement [50]. The use of game-like components in health and nutrition apps is widespread and some initial studies demonstrate that women in particular may derive social benefit from gamification [51-53]. The *Fit After Baby* program also included the engagement strategies of push notifications to remind or trigger the user to interact with the app, as well as *favoriting*, in which users could curate their own health library for future use and thereby personalize the app.

A registered dietitian with experience in both the DPP and motivational interviewing served as the lifestyle coach for the *Fit After Baby* program. She based her conversations on the weekly content contained in the app but was encouraged to follow the participants’ lead if they wanted to discuss other topics. The lifestyle coach kept detailed notes on her conversations and reviewed any issues weekly with the principal investigator.

### Iterative Feedback and Design Process

Rapid iterative design is a process in which technology is tested and repeatedly refined with small groups of users to optimize functionality and usability [54,55]. We refined the content and delivery of the mHealth *Fit After Baby* program over three rounds of beta-testing through an iterative design process, and then we developed a final version of the program.

#### Recruitment

Women between 18 and 40 years of age with a postpartum BMI of 24 to 45 kg/m^2^ who were within 6 months of a recent singleton or twin delivery complicated by gestational hypertension, preeclampsia, preterm delivery (32-37 weeks), an SGA neonate (weight <10th percentile for gestational age), and/or GDM were recruited for the study from women delivering at the University of Colorado Hospital on the Anschutz Medical Campus in Aurora, Colorado. Women were required to have access to an iPhone or iPod (Apple Inc, California) touch ≥5S, and women with a history of preexisting diabetes, cancer, or CVD were excluded. The institutional review board at the University of Colorado approved the study, and all patients gave written informed consent.

#### Pilot Testing

We conducted three 4-week beta-testing pilots with unique participants. The participants used the *Fit After Baby* app for 4 weeks, received support from the lifestyle coach, and provided ongoing feedback. Participants were required to open the app and provide online feedback in week 1 to receive a Fitbit by mail to use for the remainder of the study. Participants were asked to log on to a Web-based asynchronous user group platform each week and answer a set of questions in threaded discussions. Interactions with the app and threaded discussions were tracked by researchers. The three 4-week pilots were conducted during the time period June 2015 through February 2016.

#### Feedback and Iterative Design

After each round, we analyzed feedback using content analysis, and changes were made to the mobile app and program before conducting subsequent rounds. Consequently, each subsequent round had a new iteration of the app and program. We held two in-person group structured interviews lasting 90 min after all three rounds were completed. Participants from each round were included. Group interviews were digitally recorded and transcribed verbatim.

#### Analysis

We used an iterative and team-based process guided by qualitative content analysis [56,57]. A qualitatively trained analyst and principal investigator both inductively and deductively developed the code book. Initial codes were based on the interview guide domains, and the code book was expanded based on codes that emerged from the data. The analyst and investigator jointly reviewed and coded the threaded discussions and group interviews until no new codes were identified and there was strong code assignment agreement. All transcripts were independently read, double coded, and then merged before analysis. Any discrepancies in coding were addressed through discussion and consensus among the coders. Throughout the analytic process, the analyst and principal investigator met regularly to check new findings, discuss emergent new codes and themes, and assess the preliminary and final results. ATLAS.ti version 8.0 was used for data organization and management. Data from all rounds and group interviews were used to design a final version of the mobile app and program.

## Results

### Demographic, Tracking, and Online Feedback Data for Beta Testing

Table 1 demonstrates the characteristics of the participants who participated in beta testing. The median age for beta testing was 33 years, with a median BMI in the obese range of 31.8 kg/m^2^. The majority of participants were white, and preterm birth, pre-eclampsia, and gestational hypertension were the most common pregnancy complications.

Table 2 shows the user data for the mobile app. The beta-testers opened the app an average of 21 out of 28 days. Participants tracked a median of 14 of 21 days of suggested diet tracking on MyFitnessPal, and tracked physical activity using Fitbit or the exercise tracker within the app for a median of 16 of 21 suggested days. We recommended that participants check in with their coach and enter their weight once per week. On average, participants checked in three times over the course of the program and weighed in five times. The overall mean weight loss was 4.3 (SD 2.3) lbs over the 4-week period. The first beta pilot round included 4 women, and they were actively engaged in the program and app. Many changes were made to the app between rounds 1 and 2 in response to feedback and user data. In round 2, there was more variable participation, with some women engaging much less in the app. After we conducted the next round of iterative changes, the engagement increased again in round 3.

**Table 1:** Demographic data of participants in three rounds of beta-testing of the mobile health Fit After Baby program.

Characteristics of beta-testers		Round 1 (n=4)	Round 2 (n=13)	Round 3 (n=9)	All groups (n=26)
Age (years), median (IQR)		34 (33.3-37)	33 (31-34.5)	30 (28-33.5)	33 (30.75-34)
**Race, n (%)**					
	White	4 (100)	12 (92)	8 (89)	24 (92)
	Asian	0 (0)	1 (8)	1 (11)	2 (8)
	Hispanic or Latino	0 (0)	0 (0)	1 (11)	1(4)
BMI (kg/m²), median (IQR)		35.9 (30.2-41.1)	31.2 (29.1-40.1)	32.3 (28.6-35.9)	31.8 (30.1-40.4)
**Pregnancy complication, n (%)**					
	Gestational diabetes	1 (25)	4 (31)	2 (22)	7 (27)
	Pre-eclampsia	1 (25)	6 (46)	2 (22)	9 (35)
	Gestational hypertension	1 (25)	2 (15)	5 (56)	8 (31)
	Small-for-gestational age	0 (0)	2 (15)	2 (22)	4 (15)
	Preterm birth	1 (25)	5 (38)	2 (22)	8 (31)

**Table 2:** User data for three rounds of beta-testing of the mobile health Fit After Baby program.

Beta-testers	Round 1 (n=4)	Round 2 (n=13)	Round 3 (n=9)	All groups (n=26)
Days app launched (out of 28), median (IQR)	24 (16-27)	16 (12-24)	21 (16-25)	20.5 (13-25)
Days diet tracked (out of 21), median (IQR)	17 (12-21)	14 (0-18)	10 (0-18)	14 (0-18)
Days steps or exercise tracked, median (IQR)	25 (19-27)	12 (5-17)	19 (7-22)	16 (9-22)
Number of surveys answered, median (IQR)	11 (9-13)	10 (2-12)	9 (5-12)	10 (4-12)
Coach check ins, median, (IQR)	2 (1-3)	1 (0-4)	4 (2.5-4)	3 (1-4)
Weigh-ins, median (IQR)	4 (4-6)	6 (4-11)	5 (4-7)	5 (4-7)
Weight loss in lbs, mean (SD)	4.4 (1.6)	4.3 (2.8)	4.1 (2.0)	4.3 (2.3)

### Feedback and Iterative Development

Feedback addressed usability, navigability, content, and function, and certain themes arose which we used to develop changes in subsequent iterations of the app and program. Multimedia Appendix 1 details the iterative changes made throughout the rounds, including representative quotes associated with the changes.

### Navigation

One major theme addressed through successive iterations was navigation. The round 1 version opened to a content screen tailored for that particular day, and participants expressed that they did not know where they were in the app and they were not clear about what they needed to do each day. In response, we developed a home screen consisting of a task list. Tapping on tasks in the list navigated the participant to the screen for that task, and a checkmark automatically appeared once the participant had completed a particular task. New participants in subsequent rounds were enthusiastic about checkmarks and the improved navigation. The final navigation strategy developed after round 3 employed rotating task cards that linked with tasks in the apps and retained the function of automatically checking off completed tasks.

### Diet Tracking

We asked participants to use MyFitnessPal for diet tracking. Participants expressed some frustration with tracking of dietary intake. Participants had to download the MyFitnessPal app and open the app to record food and drink, and they complained that it was difficult to switch back and forth between apps. As has been shown previously, tracking of dietary intake is difficult and cumbersome, and can be difficult to maintain for long periods of time [58]. Several participants expressed how difficult it was to track dietary intake while taking care of an infant. In the ultimate version of the program, we elected to ask patients to track diet for only certain days coordinated with a particular dietary theme (ie, tracking saturated fat during week 6 which focuses on dietary fat). However, we retained the option to track every day and to earn points for this. In the final version, we also decided to ask participants to use the Fitbit app for both diet and exercise tracking, so they would not have to download or navigate to the MyFitnessPal app, and therefore there would be less back and forth between apps.

### Tracking and Syncing of Physical Activity

Participants were asked to use Fitbit for step tracking, and there was also an exercise tracker built into the *Fit After Baby* app. Participants were sent a Fitbit Zip at the beginning of week 2, if they had been using the app. They needed to download the Fitbit app and connect it with their Fitbit device. Although participants did not actually need to open the Fitbit app once it was downloaded, they complained that their steps were not always syncing qith the Fit AFter Baby app. The majority of participants stated that they would have preferred a wrist band Fitbit, so we transitioned to this for the final version of the program. The wrist Fitbit (Fitbit Flex 2) is also waterproof, so we felt that this would encourage adherence since some participants told us they forgot to remove the clip Fitbits from their clothes before washing them. We retained the ability to log workouts in the *Fit After Baby* app specifically to give participants the ability to keep track and earn points if they forgot to wear their Fitbit.

### Content

Participants in all three rounds requested more content, particularly content tailored specifically to postpartum women. In round 1, the most common requests for additional content included more information about breastfeeding, working, and postpartum mental health. This content was added before round 2, and group 2 also requested additional content about breastfeeding, as well as healthy recipes and information about exercising with an infant. Although we added this content, round 3 participants requested even more exercise information as well as recipes and more detailed diet information. For the final version of the program, we added additional exercise content, multiple exercises with instructions and pictures demonstrating ways to exercise with an infant, a yoga curriculum, and a more extensive recipe collection.

### Photos/Graphics

Participants from all three rounds requested more diversity in photos to better reflect their own postpartum experiences. They expressed that women who look like thin models did not reflect what it was like to be a postpartum mom struggling to lose weight. However, some women mentioned that the photos were appropriate, so at each round, we worked to diversify some images but kept others. A few women also commented that the women in the photos looked too calm and relaxed, and they wanted to see some images that would reflect the stress of parenting a newborn. In response, we added additional images to reflect the broad range of emotional experiences postpartum.

### Points and Rewards

Participants in round 1 felt that the points and badges had little meaning and it was not clear how points were earned. Before round 2, we simplified the badges and designed an explanatory function that would detail what activities earned points and how many points were earned. However, participants continued to express confusion about point accumulation and also felt that the point and reward system was not motivating. When asked, participants were enthusiastic about the idea of tangible rewards like gift cards. In our final version, we improved the points and rewards system by simplifying our point system to a single health warrior badge with 4 levels (bronze, silver, gold, and platinum). When a participant reaches each health warrior level, they earn gift cards that we send to their email.

### Coaching

In round 1, some of the participants mentioned that although they knew that they were supposed to check in with their coach, it was not clear what they were supposed to talk about. For the subsequent iterations, we added topic suggestions to each coach check-in prompt. Some participants also felt the coach check-in should not be the responsibility of the participant but should rather be initiated by the coach. Others mentioned that the information in the coaching sessions should be more individualized. Participants also felt that when the coach was more aware of exactly what participants were doing with exercise, diet, and weight, they were more motivating. To help the coach provide more targeted coaching, we designed a coach app with a dashboard showing individual activities by participants who were using the *Fit After Baby* app, dietary intake, weight, steps, and points earned. This enabled the coach to follow each participant’s activities in real time and provide targeted feedback and coaching.

### Website

We designed a companion website to the *Fit After Baby* program. The website was designed to contain additional content that was not included in the app, including recipes. In addition, the website also provided a portal for participants to view personal data and rewards in greater depth and at a larger visual size. However, the website was rarely used, and most participants stated that the website was not useful or that they did not use the website because it was not easy to use on their mobile phones. We decided to remove the companion website for the final iteration of the program.

### Additional Suggested Features

Several participants requested that mind-body and meditation techniques be included in the app. In the final iteration, we added a substantial amount of content including mind-body techniques and a yoga curriculum introducing two new poses per week. Many participants also expressed interest in sharing their information socially with the cohort. Further discussion revealed that they preferred the idea of sharing within the group simultaneously participating in the *Fit After Baby* program and going through similar challenges to sharing with friends or family members. There were mixed reviews on the idea of competition within the cohort, and on the idea of working together to earn points toward a common goal. Other participants requested more stories from women who had succeeded at achieving their postpartum goals. These features were not added to the final version but may be considered in the future.

### Overall Impression of the Program

Overall, most participants responded that they were satisfied with the *Fit After Baby* program. Interestingly, we found that although the participants responded favorably to the content in general, they consistently requested more at each round. This feedback substantially modified the content of subsequent versions and was valuable in informing the final iteration of the program. Our improvements in the navigation of the app resulted in fewer complaints about navigational issues in subsequent iterations. All participants from the third iteration who responded in week 4 about whether they would recommend the *Fit After Baby* program to others said that they would. Participants in the group interviews responded favorably to the program overall and affirmed that it was helpful. They felt that the program provided motivation and accountability to make changes in the postpartum period. Both participants in the second group interview expressed that they would have liked to continue to use the program. Overall, participants felt that the most useful components were the reminders and tracking components, and almost everyone seemed to enjoy using the Fitbit. In addition, most seemed to appreciate the information on healthy eating, quick and easy recipes, and exercises to do with baby.

## Discussion

### Principal Findings

We successfully employed an interactive, user-centered, participatory method of rapid, iterative design and evaluation to optimize the mHealth *Fit After Baby* program for postpartum women. Our multidisciplinary team of researchers and designers substantially improved the content and functionality of the app at each successive iteration by integrating user feedback. Qualitative data collected in the *Fit After Baby* development process provide valuable insight into the use of mobile technology–based weight loss apps in the target population of postpartum women at elevated cardiometabolic risk.

The most common and consistent theme through the iterative development process was a request for added content, and particularly added content tailored to postpartum mothers. Participants requested more postpartum-focused diet, exercise, and weight loss information, and also information on breastfeeding and postpartum mental health, including mind-body techniques for coping with stress. A consistent theme was the need to improve navigation because of difficulties in navigating to the appropriate screen. We responded to suggestions about navigation by creating a home screen with checkboxes so that daily and weekly tasks were more clear. The feedback from our participants suggested that the navigation was improved significantly by the final iteration. We were somewhat surprised to learn that women did not find the companion website to be useful, and furthermore, they found it difficult to use on a mobile phone. We eliminated the companion website for the final iteration. Diet and exercise tracking turned out to be challenging to most of our participants. Participants were clearly frustrated with the amount of time it took to track their diet and also disliked switching between apps, so in our final iteration, we limited tracking to particular days and decreased app switching by using the Fitbit app to track both diet and exercise. Many women said that they would prefer a wrist Fitbit, and we moved to this for the final version. We learned that the gamification component of points and badges was confusing and not very motivating. Many women said that they would prefer a tangible reward, so we added in the opportunity to earn gift cards for the final version. The participants also expressed a need for content that we did not originally include which led to the addition of a week of content focusing on mental health, anxiety, and stress in the final version.

### Limitations

There are several limitations to this study. We developed and tested the *Fit After Baby* app on an iPhone Operating System platform. This affected the demographics and economic status of the recruited participants, which may thereby affect generalizability. We are currently developing an Android version of the *Fit After Baby* app and will be able to use both versions for future studies. The group sizes for the iterations were small, and therefore may not be adequately representative. Consistent with the demands of women during this very challenging postpartum period, all participants did not contribute to the online focus group every week. Although we attempted to conduct 2 in-person focus groups, we had several women who were unable to attend at the last minute and had to convert the 2 focus groups to 2-person group interviews. For this iterative design process, we did not include a control group, and therefore are unable to assess the significance of postpartum weight loss in this study.

### Conclusions

*Fit After Baby* is a theory-based app using mHealth weight loss best practices and developed using the principles of rapid iterative design. To our knowledge, *Fit After Baby* is one of only a few weight loss apps designed specifically for postpartum women and the only one specifically focused on postpartum women at increased cardiometabolic risk. The design process involved a multidisciplinary team of researchers in collaboration with a technology team. We found the process of iterative development to substantially change the content and function of the Fit After Baby program which may be helpful for the development of similar programs in the future. Our first iteration compared with our final version has undergone substantial modification owing to this iterative process, and we are currently testing the final version of the *Fit After Baby* program in a pilot randomized trial with a primary goal of postpartum weight loss.

## Appendix

Multimedia Appendix 1 Representative quotes and iterative changes made throughout the three rounds of pilot testing and for the final version.

## Abbreviations

CVD: cardiovascular disease
DPP: Diabetes Prevention Program
GDM: gestational diabetes mellitus
mHealth: mobile health
NIH: National Institutes of Health
SGA: small-for-gestational age

## References

[1] Mosca L, Benjamin EJ, Berra K, Bezanson JL, Dolor RJ, Lloyd-Jones DM, Newby LK, Piña IL, Roger VL, Shaw LJ, Zhao D, Beckie TM, Bushnell C, D'Armiento J, Kris-Etherton PM, Fang J, Ganiats TG, Gomes AS, Gracia CR, Haan CK, Jackson EA, Judelson DR, Kelepouris E, Lavie CJ, Moore A, Nussmeier NA, Ofili E, Oparil S, Ouyang P, Pinn VW, Sherif K, Smith SC, Sopko G, Chandra-Strobos N, Urbina EM, Vaccarino V, Wenger NK (2011). Effectiveness-based guidelines for the prevention of cardiovascular disease in women--2011 update: a guideline from The American Heart Association. Circulation.

[2] Gunderson EP, Chiang V, Pletcher MJ, Jacobs DR, Quesenberry CP, Sidney S, Lewis CE (2014). History of gestational diabetes mellitus and future risk of atherosclerosis in mid-life: the Coronary Artery Risk Development in Young Adults study. J Am Heart Assoc.

[3] Tobias DK, Hu FB, Forman JP, Chavarro J, Zhang C (2011). Increased risk of hypertension after gestational diabetes mellitus: findings from a large prospective cohort study. Diabetes Care.

[4] Rich-Edwards JW (2012). The predictive pregnancy: what complicated pregnancies tell us about mother's future cardiovascular risk. Circulation.

[5] Fraser A, Nelson SM, Macdonald-Wallis C, Cherry L, Butler E, Sattar N, Lawlor DA (2012). Associations of pregnancy complications with calculated cardiovascular disease risk and cardiovascular risk factors in middle age: the Avon Longitudinal Study of Parents and Children. Circulation.

[6] Villamor E, Cnattingius S (2006). Interpregnancy weight change and risk of adverse pregnancy outcomes: a population-based study. Lancet.

[7] Rodríguez-González Guadalupe L, Castro-Rodríguez Diana C, Zambrano Elena (2018). Pregnancy and Lactation: A Window of Opportunity to Improve Individual Health. Methods Mol Biol.

[8] Bentley-Lewis R, Levkoff S, Stuebe A, Seely EW (2008). Gestational diabetes mellitus: postpartum opportunities for the diagnosis and prevention of type 2 diabetes mellitus. Nat Clin Pract Endocrinol Metab.

[9] Smith GN (2009). Development of preeclampsia provides a window of opportunity for early cardiovascular risk screening and intervention. Expert Rev Obstet and Gynecol.

[10] Knowler WC, Barrett-Connor E, Fowler SE, Hamman RF, Lachin JM, Walker EA, Nathan DM, Diabetes Prevention Program Research Group (2002). Reduction in the incidence of type 2 diabetes with lifestyle intervention or metformin. N Engl J Med.

[11] Eckel RH, Jakicic JM, Ard JD, de Jesus JM, Miller NH, Hubbard VS, Lee I, Lichtenstein AH, Loria CM, Millen BE, Nonas CA, Sacks FM, Smith SC, Svetkey LP, Wadden TA, Yanovski SZ, Kendall KA, Morgan LC, Trisolini MG, Velasco G, Wnek J, Anderson JL, Halperin JL, Albert NM, Bozkurt B, Brindis RG, Curtis LH, DeMets D, Hochman JS, Kovacs RJ, Ohman EM, Pressler SJ, Sellke FW, Shen W, Smith SC, Tomaselli GF, American College of Cardiology/American Heart Association Task Force on Practice Guidelines (2014). 2013 AHA/ACC guideline on lifestyle management to reduce cardiovascular risk: a report of the American College of Cardiology/American Heart Association Task Force on Practice Guidelines. Circulation.

[12] Bao W, Tobias DK, Bowers K, Chavarro J, Vaag A, Grunnet LG, Strøm M, Mills J, Liu A, Kiely M, Zhang C (2014). Physical activity and sedentary behaviors associated with risk of progression from gestational diabetes mellitus to type 2 diabetes mellitus: a prospective cohort study. JAMA Intern Med.

[13] Hedderson M, Ferrara A (2014). A call to increase physical activity among women of reproductive age: is it possible?. JAMA Intern Med.

[14] Huang Z, Willett WC, Manson JE, Rosner B, Stampfer MJ, Speizer FE, Colditz GA (1998). Body weight, weight change, and risk for hypertension in women. Ann Intern Med.

[15] Catalano PM, Ehrenberg HM (2006). The short- and long-term implications of maternal obesity on the mother and her offspring. Br J Obstet Gynaecol.

[16] Glazer NL, Hendrickson AF, Schellenbaum GD, Mueller BA (2004). Weight change and the risk of gestational diabetes in obese women. Epidemiology.

[17] Hoedjes M, Berks D, Vogel I, Franx A, Duvekot JJ, Oenema A, Steegers EA, Raat H (2012). Motivators and barriers to a healthy postpartum lifestyle in women at increased cardiovascular and metabolic risk: a focus-group study. Hypertens Pregnancy.

[18] Østbye T, Krause KM, Lovelady CA, Morey MC, Bastian LA, Peterson BL, Swamy GK, Brouwer RJ, McBride CM (2009). Active Mothers Postpartum: a randomized controlled weight-loss intervention trial. Am J Prev Med.

[19] Sarwer DB, Allison KC, Gibbons LM, Markowitz JT, Nelson DB (2006). Pregnancy and obesity: a review and agenda for future research. J Womens Health (Larchmt).

[20] Nicklas JM, Barbour LA (2015). Optimizing weight for maternal and infant health - tenable, or too late?. Expert Rev Endocrinol Metab.

[21] Jones EJ, Peercy M, Woods JC, Parker SP, Jackson T, Mata SA, McCage S, Levkoff SE, Nicklas JM, Seely EW (2015). Identifying postpartum intervention approaches to reduce cardiometabolic risk among American Indian women with prior gestational diabetes, Oklahoma, 2012-2013. Prev Chronic Dis.

[22] Dasgupta K, da Costa D, Pillay S, de Civita M, Gougeon R, Leong A, Bacon S, Stotland S, Chetty VT, Garfield N, Majdan A, Meltzer S (2013). Strategies to optimize participation in diabetes prevention programs following gestational diabetes: a focus group study. PLoS One.

[23] Hearn L, Miller M, Lester L (2014). Reaching perinatal women online: the Healthy You, Healthy Baby website and app. J Obes.

[24] Smith A (2015). Pew Research Center.

[25] Breton ER, Fuemmeler BF, Abroms LC (2011). Weight loss-there is an app for that! But does it adhere to evidence-informed practices?. Transl Behav Med.

[26] Khaylis A, Yiaslas T, Bergstrom J, Gore-Felton C (2010). A review of efficacious technology-based weight-loss interventions: five key components. Telemed J E Health.

[27] Pagoto S, Schneider K, Jojic M, DeBiasse M, Mann D (2013). Evidence-based strategies in weight-loss mobile apps. Am J Prev Med.

[28] Azar KM, Lesser LI, Laing BY, Stephens J, Aurora MS, Burke LE, Palaniappan LP (2013). Mobile applications for weight management: theory-based content analysis. Am J Prev Med.

[29] Conroy DE, Yang C, Maher JP (2014). Behavior change techniques in top-ranked mobile apps for physical activity. Am J Prev Med.

[30] Burke LE, Ma J, Azar KM, Bennett GG, Peterson ED, Zheng Y, Riley W, Stephens J, Shah SH, Suffoletto B, Turan TN, Spring B, Steinberger J, Quinn CC, American Heart Association Publications Committee of the Council on Epidemiology and Prevention‚ Behavior Change Committee of the Council on Cardiometabolic Health‚ Council on Cardiovascular and Stroke Nursing‚ Council on Functional Genomics and Translational Biology‚ Council on Quality of Care and Outcomes Research‚Stroke Council (2015). Current science on consumer use of mobile health for cardiovascular disease prevention: a scientific statement from The American Heart Association. Circulation.

[31] Mummah SA, King AC, Gardner CD, Sutton S (2016). Iterative development of Vegethon: a theory-based mobile app intervention to increase vegetable consumption. Int J Behav Nutr Phys Act.

[32] Ybarra ML, Prescott TL, Philips GL, Bull SS, Parsons JT, Mustanski B (2016). Iteratively developing an mHealth HIV prevention program for sexual minority adolescent men. AIDS Behav.

[33] Lin PH, Intille S, Bennett G, Bosworth HB, Corsino L, Voils C, Grambow S, Lazenka T, Batch BC, Tyson C, Svetkey LP (2015). Adaptive intervention design in mobile health: intervention design and development in the Cell Phone Intervention for You trial. Clin Trials.

[34] DiFilippo KN, Huang W, Chapman-Novakofski KM (2017). A New Tool for Nutrition App Quality Evaluation (AQEL): development, validation, and reliability testing. JMIR Mhealth Uhealth.

[35] Bull S, Ezeanochie N (2016). From Foucault to Freire through Facebook: toward an integrated theory of mHealth. Health Educ Behav.

[36] Webb TL, Sniehotta FF, Michie S (2010). Using theories of behaviour change to inform interventions for addictive behaviours. Addiction.

[37] Ajzen I, Kuhl J, Beckmann J (1985). From intentions to actions: a theory of planned behavior. Action-Control: From Cognition to Behavior.

[38] Lu MC, Kotelchuck M, Culhane JF, Hobel CJ, Klerman LV, Thorp JM (2006). Preconception care between pregnancies: the content of internatal care. Matern Child Health J.

[39] Beckles GL, Thompson-Reid PE (2001). CDC stacks.

[40] Bandura A (1997). Self-Efficacy: The Exercise of Control.

[41] van Gemert-Pijnen JE, Nijland N, van Limburg M, Ossebaard HC, Kelders SM, Eysenbach G, Seydel ER (2011). A holistic framework to improve the uptake and impact of eHealth technologies. J Med Internet Res.

[42] Eysenbach G (2008). Medicine 2.0: social networking, collaboration, participation, apomediation, and openness. J Med Internet Res.

[43] Riley WT, Rivera DE, Atienza AA, Nilsen W, Allison SM, Mermelstein R (2011). Health behavior models in the age of mobile interventions: are our theories up to the task?. Transl Behav Med.

[44] Whittaker R, Merry S, Dorey E, Maddison R (2012). A development and evaluation process for mHealth interventions: examples from New Zealand. J Health Commun.

[45] Zhang J (2005). Human-centered computing in health information systems. Part 1: analysis and design. J Biomed Inform.

[46] Hingle M, Patrick H (2016). There are thousands of apps for that: navigating mobile technology for nutrition education and behavior. J Nutr Educ Behav.

[47] Waterlander W, Whittaker R, McRobbie H, Dorey E, Ball K, Maddison R, Smith KM, Crawford D, Jiang Y, Gu Y, Michie J, Mhurchu CN (2014). Development of an evidence-based mHealth weight management program using a formative research process. JMIR Mhealth Uhealth.

[48] Michie S, Abraham C, Whittington C, McAteer J, Gupta S (2009). Effective techniques in healthy eating and physical activity interventions: a meta-regression. Health Psychol.

[49] Stephens J, Allen JK, Himmelfarb CR (2011). 'Smart' coaching to promote physical activity, diet change, and cardiovascular health. J Cardiovasc Nurs.

[50] Hickey DT (2012). The EvoLLLution.

[51] Koivisto J, Hamari J (2014). Demographic differences in perceived benefits from gamification. Comput Human Behav.

[52] Miller AS, Cafazzo JA, Seto E (2016). A game plan: Gamification design principles in mHealth applications for chronic disease management. Health Informatics J.

[53] Lister C, West JH, Cannon B, Sax T, Brodegard D (2014). Just a fad? Gamification in health and fitness apps. JMIR Serious Games.

[54] Gary K, Enquobahrie A, Ibanez L, Cheng P, Yaniv Z, Cleary K, Kokoori S, Muffih B, Heidenreich J (2011). Agile methods for open source safety-critical software. Softw Pract Exp.

[55] Larman C, Basili VR (2003). Iterative and incremental developments. A brief history. Comput.

[56] Hsieh H, Shannon SE (2005). Three approaches to qualitative content analysis. Qual Health Res.

[57] Stemler S (2000). An overview of content analysis. Pract Assess Res Eval.

[58] Burke LE, Warziski M, Starrett T, Choo J, Music E, Sereika S, Stark S, Sevick MA (2005). Self-monitoring dietary intake: current and future practices. J Ren Nutr.

