# Supplementary material for: Development and Modification of a Mobile Health Program to Promote Postpartum Weight Loss in Women at Elevated Risk for Cardiometabolic Disease: Single-Arm Pilot Study
Source: JMIR Form Res. 2020 Apr 9;4(4):e16151. doi: 10.2196/16151 (PMC7180508; doi:10.2196/16151)
Supplement: Multimedia Appendix 1 [file formative_v4i4e16151_app1.docx]

| **Feedback Topic** | **Round^a^** | **Representative quote** | **Iterative Action taken** |
| --- | --- | --- | --- |
| **Navigation** | 1 | “The content has been interesting but the layout doesn’t flow well. I think that when you open the app, the content for that particular day should be present and you should not need to go to a drop down menu to find it.” (FAB06) | Task list screen created:  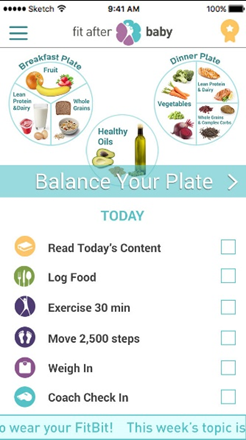 |
|  | 2 | “Overall, I would prefer that tasks were in some way checked off once completed, as it gives more of a sense of accomplishment and encourages one to keep tracking.” (FAB19) |  |
|  | 3 | “…I love the check marks only because as a mom of 4 it is hard to remember what has been completed, so this helps me stay on track on a daily basis.” (FAB22) |  |
| **Diet Tracking** | 1 | “I did not like the fact that I felt like I was constantly logging in to apps to log everything I ate or drank or every time I moved. This leads to a lot of screen time to an already busy and overtired life.” (FAB04) | Decreased the number of days participants were asked to track diet.  Changed diet tracking app to Fitbit from My Fitness Pal to decrease back and forth between apps. |
|  | 2 | “I know it’s helpful for losing weight and staying accountable, but honestly, it is way inconvenient when trying to take care of a newborn baby (babies in my case). Trying to fit in exercise, a shower, household tasks, AND feed/pump/clean, tracking calories is the last thing I want to do…” (FAB13) |  |
|  | 3 | “I'm not sure about the tracking. I do think I think more about what I'm eating when I know it's being tracked but I also don't really like having to log every meal every day with everything else going on!” (FAB24) |  |
| **Physical Activity Tracking** | 1 | “I agree with other users that I felt like I was triple entering data in FAB, fitness pal and Fitbit. This shouldn’t have been necessary and they should have been more easily linking.” (FAB02) | For the final version of the program we had participants do both diet and exercise tracking in Fitbit, with an option to manually record in the FAB app if needed. |
|  | 3 | “One thing is I often forgot Fitbit when I changed clothes. You don’t really change clothes on a schedule, it’s like ok its 3 o’clock so I’ll put on something else. And then I would feel defeated because all the steps wouldn’t get counted because I’d leave the Fitbit on my stupid pajama pants. It did motivate me to – one of my aunts was done with her Fitbit for her wrist so I got that. Then when I get up all night I remember I have it on. Thought it was visually good and a reminder to ask someone to stay here while I go on a quick walk.” (FAB29-GI2) | For the final version of the FAB program we switched from a clip on Fitbit to a wrist Fitbit. |
| **Content/**  **Tailoring** | 1 | “For me it needs to be highly structured on postpartum women. I like science, I want to know physically why and know why my body is changing the way that it is. I feel like I can go anywhere to get advice on stress management and exercise but needs to be specific. Specific like, during the first month after delivery… How has that changed and how it is so different? And I want that specific information. I know about fitness and health, but I haven’t had a baby before. I want to know about women’s bodies changing and when it will go back to normal or will it go back to normal.” (FAB52-GI1)  “I think that topics on how to balance being a working mother with raising a child may be better received. I also think that adding some content about breastfeeding nutrition may be helpful for those who are breastfeeding.” (FAB04)  “…more postpartum-specific information (i.e. Exercise to strengthen a c-section belly, how to motivate yourself when you feel you are already pushed to your limits with stress, fatigue, etc.)” (FAB52) | Added content:  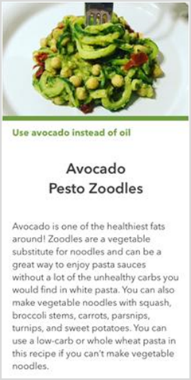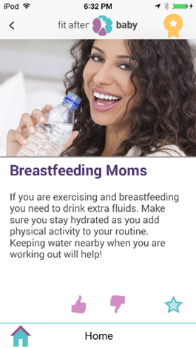  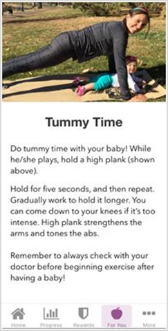 |
|  | 2 | “Definitely would like to see workouts, stress relief, something about the lack of or broken sleep, and healthy recipes that are easy to make.” (FAB07)  “Healthy eating options and ways to exercise with 2 kids in tow.” (FAB18) |  |
|  | 3 | “…more exercise info would have been helpful (like safe exercises, etc). An easy exercise of the day would have been great!! Especially things that you can do while multi-tasking (so maybe it's lunges while on your walk or squats while holding baby, etc.)” (FAB28) |  |
| Photos/  Graphics | 1 | “I’m not sure they [photos] could be more personal or targeted to me, other than not putting very slim women on the app when I certainly do not feel that way. Maybe ‘normalize’ the weight loss struggle by adding photos of women who are also trying to lose weight, instead of very thin models.” (FAB52) | Replaced some photos with more diverse body sizes:  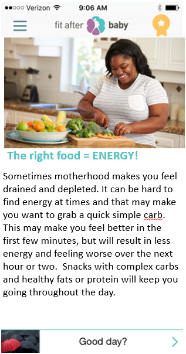 |
|  | 2 | “I thought the pictures were good. I think the pictures should reflect women who have just given birth. Not everyone gets their body back right away and may still be bigger/ heavier set, seeing pictures of skinnier women made me feel like I wasn’t doing anything right since I still haven’t lost my baby weight or got my body back.” (FAB07)  “…I liked the images and felt that they were appropriate.” (FAB53) |  |
|  | 3 | “They looked like very well put together and happy people not something that actively reflects stressed out, overweight, working parents with newborns....Haha! Otherwise I felt that they were acceptable images.” (FAB21) | Added photos to reflect variety of emotional experiences:  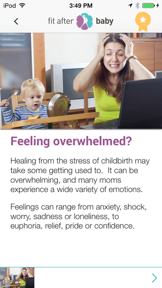 |
| Points and Rewards | 1 | “I initially felt motivated by the rewards but very quickly realized I had no idea what they meant. As a result they became far less motivating.” (FAB02) | Streamlined reward system to earning points toward levels:  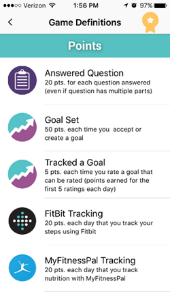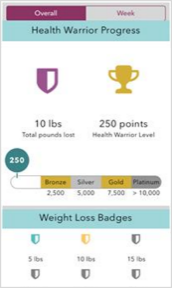 |
|  | 2 | “The points system as it is now is not very motivating – I think if there were levels that could be more helpful. The total number of points I had didn’t seem to mean much overall.” (FAB19) |  |
|  | 3 | “I would be more motivated for the badges for points if there was some sort of competition or realistic reward I was working for.” (FAB21) | Added gift card rewards for reaching levels. |
| Coaching | 1 | “I would like more input form the coach on my specific goals/weight loss/nutrition and progress, as well as tips on how to improve.” (FAB04)  “It should not be left up to the participant to determine how often coaching session take place…I think the coach would be most helpful by providing individualized advice, since every participant has unique challenges and barrier to goals that the app can’t possibly know.” (FAB52) | We added more structure to the coach check-ins by suggesting topics.  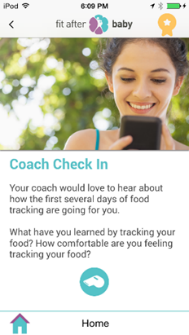 |
|  | 2 | “I think checking in with the coach was helpful, both for having that sense of responsibility and wanting to perform well for them, as well as getting more personalized feedback from them.” (FAB19) | We developed a separate coach app so that she could easily visualize progress and provide targeted coaching:  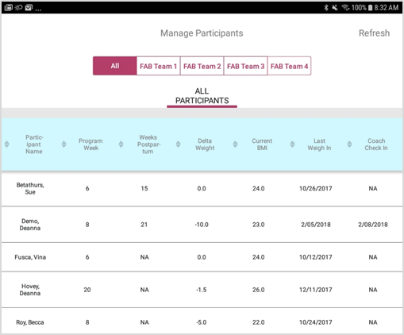 |
|  | 3 | “I would have liked more communication like this from my coach. I wasn't sure if the coach was really looking at my progress regularly or just when I emailed (or at all), so knowing that they were checking in on me may have motivated me more & made me feel more supported. I thought the coach was reviewing my diet, which made me make different/better choices when I was tracking my calories. I think a review of diet with suggestions would have been helpful.” (FAB28) |  |
| Website | 1 | “I find it cumbersome to log onto a website and use an app. That is what apps were designed for, to avoid the websites.” (FAB04) | Removed website from program. |
|  | 3 | “I have not searched through the website much… I find it easier to use the app. I do not always have access to a computer or laptop and always have my phone with me. The app is quite easy as I mentioned before but if the website doesn’t work well on a smart phone then I am not likely to use it.” (FAB50)  “And at the point you’re going to a website, it just kind of opens up like, why go to this website that’s pretty repetitive of what we’ve already seen on our phones so instead I might go look at something else.” (FAB29-GI2) |  |
| Additional suggested features | 1 | “Powerful hearing other mom’s stories. How other moms lost weight and want to know how they did that. What are they doing and why did it work. Seeing things like this woman has twins and I only had one but she did it.” (FAB52-GI1) | We are hoping to add personal stories to a future iteration of the Fit After Baby program. |
|  | 2 | “Specific workouts for the day, or maybe stress management suggestions, like recommending trying meditation.” (FAB19) | 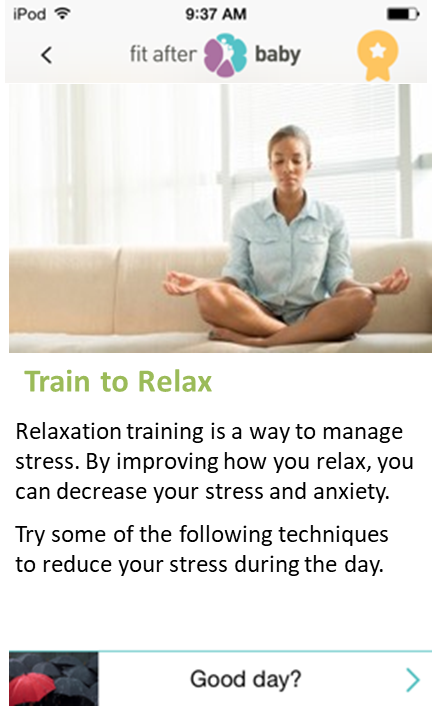  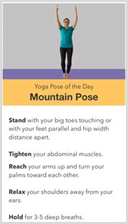 |
|  | 2 | “I think it would have been good for a small team of us to get together and work out, especially for us first time moms, get to know people, have people who experienced the same thing as you and to push you and motivate you. I wouldn’t have liked the competing as we all were on different fitness levels before giving birth so those of us who were not as active may feel like we aren’t getting our bodies back or able to do things others can and that can make someone feel down/upset about it…I would have liked that, especially sharing workouts or recipes.” (FAB07) | We are still exploring how best to integrate a social component into the Fit After Baby program. |
|  | 3 | “I think I would rather create a community with fellow moms, rather than with family and friends involved in this. Partially because I feel like the kinds of things that women struggle with postpartum aren’t the kinds of things that I necessarily want to be sharing with my friends and family.” (FAB28-GI2) |  |
|  | 3 | “I think it would be good to have the overall group split in 2 and those groups work together while also competing against the other group. This would allow more help and support from other moms and not just the coach.” (FAB30) |  |
| Overall Impression | 3 | “I really liked it. The reminders and goal of exercise helped. Big fear of maternity leave was I was just going to sit here all day. It’s a lot to get the stroller downstairs and get the dog and the car seat so I liked having the external motivation to get up and do something. Very manageable amounts of exercise, no one was asking you to run a marathon. It was just having that ping of a reminder to go exercise and that is what I liked about it. I disliked the meal tracking most. I have never tracked food before and grew up with a lot of really bad views of food and health and weight and those things being correlated. I just have never calorie counted before so I had a lot of hesitation about it.” (GI2-FAB28) |  |

Multimedia Appendix: Representative quotes and iterative changes made throughout the three rounds of pilot testing and for the final version

^a^ Participants participating in round 1,2 or 3 of online threaded discussion or participating in group interview (GI) 1 or 2
